# A comparison between bacterial cultivation and 16S rRNA next generation sequencing approaches for analysis of bacteria in urine and cerebrospinal fluid samples

**DOI:** 10.1371/journal.pone.0350939

**Published:** 2026-06-25

**Authors:** Rasmi Abu-Helu, Amal Abuhilal, Yasmin Attili, Murad Ibrahim, Ghaleb Adwan, Ibrahim Abbasi

**Affiliations:** 1 Department of Medical Laboratory Sciences, Faculty of Health Professions, Al-Quds, University, East Jerusalem, Palestine; 2 Al Quds-Bard College, Al-Quds University, East Jerusalem, Palestine; 3 Department of Microbiology and Immunology, Faculty of Medicine, Al-Quds University, East Jerusalem, Palestine; 4 An-Najah National University, Nablus, Palestine; Cornell University, UNITED STATES OF AMERICA

## Abstract

Accurate identification of bacterial pathogens is essential for effective clinical management; however, bacterial diagnosis remains challenging in routine microbiology laboratories, particularly in emergency settings and outbreak situations. Bacterial meningitis is a life-threatening condition associated with high morbidity and mortality, underscoring the need for sensitive and specific diagnostic approaches. Conventional culture-based methods often fail to identify causative agents, especially in samples with low bacterial load or fastidious organisms. This study aimed to evaluate next-generation sequencing (NGS) as a diagnostic tool compared with classical cultivation methods for bacterial detection in urine and cerebrospinal fluid (CSF) samples. A total of 50 urine samples (30 positive, 15 insignificant, and 5 negative by culture) and 14 CSF samples (4 positive and 10 negative) were collected from the microbiology laboratory at Al-Makassed Hospital in Jerusalem, Palestine. Bacterial identification was performed using both conventional methods and NGS targeting the 16S rRNA gene. Classical culture methods identified common uropathogens, including *Escherichia coli*, *Klebsiella* spp., *Enterococcus* spp., *Lactobacillus* spp., Group B *Streptococcus*, *Acinetobacter* spp., and *Proteus mirabilis* in urine samples, while CSF cultures detected coagulase-negative *Staphylococcus*, *Enterobacter* spp., and methicillin-resistant *Staphylococcus aureus* (MRSA). In comparison, NGS provided broader bacterial profiling at genus and species levels. In urine samples with significant growth, NGS identified *Escherichia coli* as the most prevalent species and *Enterococcus* as the most abundant genus. Importantly, NGS detected multiple bacterial taxa in samples classified as culture-negative or insignificant-growth, including *Escherichia*, *Enterococcus*, *Pseudomonas*, *Staphylococcus*, and *Klebsiella*. In CSF samples, NGS additionally identified *Pseudomonas*, *Enterobacter*, *Escherichia*, *Staphylococcus*, and *Rickettsia* in culture-negative specimens. Overall, NGS demonstrated substantially higher sensitivity than classical culture methods and provided more comprehensive bacterial identification, highlighting its diagnostic value for detecting slow-growing, fastidious, and low-abundance bacteria missed by conventional techniques.

## 1. Introduction

Microorganisms exist as complex, multispecies communities rather than in isolation. In humans, diverse microbial populations colonize multiple anatomical sites and interact within polymicrobial biofilms [1]. This complexity presents substantial challenges for clinical microbiology, where accurate identification of pathogenic organisms is essential for effective patient management and improved clinical outcomes [2]. Conventional diagnostic approaches frequently fail to detect causative agents, particularly in polymicrobial or low-burden infections. Advances in genome sequencing technologies, especially next-generation sequencing (NGS), offer culture-independent strategies for comprehensive microbial detection directly from clinical specimens [2].

Culture-based methods remain central to diagnostic microbiology, as they enable phenotypic characterization, including antimicrobial susceptibility testing, which is critical for guiding therapy [3]. However, cultivation is labor-intensive, technically demanding, and often limited in sensitivity, particularly for fastidious or slow-growing organisms [4]. The increasing incidence of invasive fungal and opportunistic infections further highlights the limitations of conventional diagnostics and the need for more sensitive and inclusive detection methods [5]. Due to its high throughput and decreasing cost, NGS has become widely adopted in microbiome research and is increasingly evaluated for clinical diagnostic applications [6].

The limitations of traditional culture methods have been underscored by studies demonstrating recovery of previously undetected organisms using modified media and molecular approaches. DNA-based identification is particularly valuable for organisms that are difficult to culture due to slow growth, limited metabolic activity, or inconsistent phenotypic characteristics. As a result, culture-independent methods are increasingly applied to improve etiological diagnosis, enhance outbreak investigations, detect viable but non-cultivable (VBNC) organisms, and identify novel or unexpected pathogens [7].

Conventional culture methods and targeted molecular assays, including PCR and multiplex real-time PCR panels, remain essential tools in clinical microbiology laboratories for the diagnosis of infectious diseases (Yoo IY et al., 2020). While culture-based techniques enable pathogen isolation and antimicrobial susceptibility testing, they are often limited by prolonged turnaround times and reduced sensitivity, particularly in patients receiving prior antimicrobial therapy or in infections caused by fastidious organisms. In contrast, molecular assays provide rapid and highly sensitive detection of predefined bacterial pathogens; however, their diagnostic scope is restricted to organisms specifically included in the assay design. Next-generation sequencing (NGS)-based microbiome analysis, particularly 16S rRNA gene sequencing, offers a broader, culture-independent, and hypothesis-free approach capable of identifying diverse bacterial taxa, including uncultivable, rare, or unexpected organisms [8]. Despite these advantages, NGS-based approaches require more complex bioinformatic analysis, longer processing times, and careful interpretation because of the potential detection of contaminants, commensal flora, or non-viable microorganisms [9]. Therefore, integrating conventional culture methods with molecular and NGS-based microbiome approaches may improve pathogen detection and enhance clinical interpretation, particularly in challenging specimens such as urine and cerebrospinal fluid (CSF) samples [10].

Technological advances in sequencing have facilitated these developments. Sanger sequencing typically generates read lengths of approximately 700–900 base pairs (bp), depending on template quality, primer design, and sequencing chemistry. High-quality reads are commonly reported around 750 bp under standard laboratory conditions [11, 12]. and was instrumental in early genomic studies, including the first human genome assembly [13]. Subsequent innovations, such as shotgun sequencing, combined sequencing and computational analysis to increase throughput [14]. Current NGS platforms are now available as benchtop instruments [12, 15, 16] and allow parallel sequencing of mixed microbial populations within a single sample [17]. Nevertheless, the short read lengths generated by most NGS platforms (50–500 bp) present challenges for taxonomic resolution and genome assembly. Adequate sequencing depth and coverage are therefore essential for accurate detection of fastidious, obligate anaerobic, and VBNC organisms [18, 19].

The 16S rRNA gene is one of the most widely used molecular markers for bacterial identification and phylogenetic analysis. The gene is approximately 1500 bp in length and consists of nine conserved regions interspersed with nine hypervariable regions (V1–V9). The conserved regions enable the design of universal primers, whereas the hypervariable regions provide species- and genus-level taxonomic discrimination among bacteria. Due to these characteristics, 16S rRNA gene sequencing has become a standard approach for microbial classification and diversity studies [20]. Illumina sequencing platforms employ sequencing-by-synthesis technology to generate large numbers of short reads simultaneously [15]. The V4 hypervariable region (~250 bp) is particularly suitable for Illumina MiSeq sequencing, as it allows complete overlap of paired-end reads, resulting in reduced error rates and improved accuracy compared with partially overlapping regions such as V3–V4 or V4–V5, albeit with some loss of discriminatory power [21]. The clinical utility of NGS is further enhanced by advanced bioinformatics pipelines that enable interpretation of complex sequencing data [22].

Urinary tract infections (UTIs) represent one of the most common bacterial infections encountered in clinical practice. Increasing antimicrobial resistance and the emergence of multidrug-resistant (MDR) organisms have reduced the effectiveness of empirical therapy [23]. UTIs may be caused by Gram-negative and Gram-positive bacteria, as well as fungi and parasites [24]. Conventional diagnostic criteria define significant bacteriuria as ≥10⁵ CFU/mL in midstream urine cultures, although lower thresholds, such as 10³ CFU/mL for Escherichia coli, may also be clinically relevant [25]. NGS-based studies have challenged the long-standing assumption of urinary sterility by demonstrating the presence of diverse, often non-cultivable microbial communities in bladder urine [25, 26].

Acute bacterial meningitis is a medical emergency characterized by rapid progression, outbreak potential, and high morbidity and mortality [27]. Prompt pathogen identification is essential to guide appropriate antimicrobial therapy and limit unnecessary broad-spectrum antibiotic use, which contributes to resistance. Many community-acquired infections are caused by organisms susceptible to narrow-spectrum antibiotics, and a substantial proportion of suspected bacterial cases are non-bacterial [28].NGS enables simultaneous detection of bacteria, viruses, fungi, and parasites in a single assay and has shown promise in the diagnosis of central nervous system infections, including meningitis, encephalitis, and myelitis. However, widespread clinical implementation remains limited by cost, accessibility, and turnaround time [29]

Diagnosis of bacterial meningitis traditionally relies on cerebrospinal fluid (CSF) analysis and culture, with infected samples typically showing neutrophilic pleocytosis, elevated protein levels, reduced glucose concentrations, positive Gram staining, and bacterial growth on appropriate media [30]. Recent studies have demonstrated that combining CSF NGS with conventional diagnostic methods, including serological testing, provides the highest diagnostic yield [31]. In contrast, rapid latex agglutination and immunological assays lack sensitivity at bacterial concentrations below 10⁵ CFU/mL, which occur in up to 45% of meningitis cases [32].

In this context, the present study aimed to compare cultivation-based diagnostics with 16S rRNA amplicon sequencing for the characterization of pathogenic bacterial communities in urine and cerebrospinal fluid samples, and to assess the added diagnostic value of NGS in routine clinical microbiology practice.

## 2. Materials and methods

### 2.1. Sample collection

Clinical samples, including urine and cerebrospinal fluid (CSF), were collected from Al-Makassed Hospital in Jerusalem, Palestine. Samples from all participants were collected between 3/04/2022 and 31/07/2022. The study was approved by the Al-Quds University Research Ethics Committee (Ref. No. 217/REC/2022; approval date: 31/01/2022). Written informed consent was obtained from all participants prior to enrollment.

A total of 50 urine samples were collected under sterile conditions, of which 30 showed positive bacterial growth, 15 showed insignificant growth, and 5 showed no bacterial growth (negative). For each urine sample, 3 mL was collected using a urine transport tube, sterile wide-mouth container, or sterile screw-cap tube. Samples were processed either immediately or after refrigeration for up to 24 hours.

Fourteen CSF samples were also collected, including 4 samples with positive bacterial growth and 10 with no growth. CSF samples were collected in sterile tubes, with volumes of approximately 1 mL from adults and children and 0.5 mL from neonates. Samples were processed immediately upon collection without refrigeration. Portions of CSF specimens were used for cytological and chemical examinations; when positive results were obtained, the remaining volume was used for microbiological analysis.

### 2.2. Classical culture analysis

All collected samples were examined for bacterial presence using standard culture-based techniques [33]. Samples were classified as positive, insignificant, or negative based on colony counts. Urine culture results were interpreted according to standard clinical microbiology guidelines. Bacterial growth ≥10^5^ CFU/mL was considered significant for urinary tract infection, while counts between 10³–10^5^ CFU/mL were interpreted in the context of clinical presentation. Growth below 10³ CFU/mL was defined as non-significant (insignificant growth) for the purposes of this study., and samples showing no growth were considered negative.

CSF samples were processed under a biosafety cabinet. Sample volume and macroscopic appearance were recorded. Cloudy specimens were subjected to Gram staining prior to centrifugation. Following centrifugation, sediments were cultured on enrichment media, including blood agar plates and chocolate agar, to support the growth of fastidious organisms. Gram staining was also performed using the remaining sediment.

### 2.3. DNA extraction, PCR and DNA amplification of 16S rRNA microbiome

Genomic DNA was extracted using a phenol–chloroform extraction followed by ethanol precipitation. Urine and CSF samples were first centrifuged to pellet bacterial cells. The pellets were resuspended in 300 µL of lysis buffer (50 mM NaCl, 10 mM EDTA, 50 mM Tris-HCl, pH 7.4, 1% Triton X-100), and 20 µL of 10 mg/mL Proteinase K (Sigma-Aldrich, USA) was added. The tubes were incubated at 60 °C for 2 hours. DNA was then purified by phenol extraction and precipitated with ethanol according to standard procedures {Green, 2012 #85}. Extracted DNA was stored at −20°C until further analysis. Bacterial detection was performed using universal 16S rRNA gene primers targeting the V3–V4 region, yielding an amplicon of approximately 460 bp from the full-length (~1500 bp) 16S rRNA gene [34].

The forward primer sequence used was 5′-CCTACGGGNGGCWGCAG-3′, and the reverse primer sequence was 5′-AGGACTACHVGGGTATCTAATCC-3′. Each primer was linked to an Illumina adaptor sequence to facilitate index addition and next-generation sequencing (NGS). The forward adaptor sequence was 5′-TCGTCGGCAGCGTCAGATGTGTATAAGAGACAG-3′, and the reverse adaptor sequence was 5′-GTCTCGTGGGCTCGGAGATGTGTATAAGAGAC-3′. PCR amplification was carried out under the following conditions: 35 cycles of denaturation at 95°C for 30 seconds, annealing at 60°C for 30 seconds, and extension at 72°C for 60 seconds, followed by a final extension at 72°C for 10 minutes. The number of amplification cycles (35 cycles) and annealing temperature (60°C) were optimized for low-biomass clinical samples (urine and CSF) to improve sensitivity and specificity compared with the standard Illumina protocol.

### 2.4. High throughput DNA deep sequencing using Illumina MiSeq platform

High-throughput sequencing was performed using the Illumina MiSeq platform in accordance with the Nextera 16S Metagenomic Sequencing Library Preparation protocol (Illumina, USA).

Two PCR reactions were conducted for each sample. The first PCR was used to amplify the bacterial 16S rRNA gene, and the second PCR was used to add dual barcode indices to enable identification of individual samples within pooled libraries. The index PCR was performed using an 8-cycle thermocycling protocol. After indexing, all urine and CSF samples were pooled and sequenced using the Illumina MiSeq system. Negative controls were included at all stages of the PCR workflow to monitor potential contamination. For each batch of PCR reactions, no-template controls were processed under identical conditions as the study samples. After amplification, all negative PCR controls were pooled into two separate samples and subjected to next-generation sequencing alongside the clinical specimens. These controls were evaluated to assess background contamination.Sequencing outputs, including those from negative controls, were generated in FASTQ format and have been made publicly available via the UseGalaxy platform (https://usegalaxy.eu/u/alquds/h/microbiome-analysis-abu-helu-et-al-2025).

### 2.5. Preparation of MiSeq DNA amplicon library

Two PCR amplification steps were performed for each sample. The first PCR was used to amplify the bacterial 16S rRNA gene (V3–V4 region), and the second PCR was used to attach dual-index barcodes for sample identification and multiplexing.

Indexing was performed using the Illumina Nextera XT Index Kit (Illumina, USA), following the manufacturer’s instructions for 16S metagenomic library preparation (Illumina, 2013), with minor adaptations for low-biomass clinical samples (urine and CSF). The index PCR consisted of 8 amplification cycles as recommended by the protocol. Purification of PCR products after both amplification steps was performed using AMPure XP magnetic beads (Beckman Coulter, USA) according to the manufacturer’s instructions. The sequencing library consisted of 66 PCR products, including 50 urine samples, 14 CSF samples, and 2 pooled negative controls that were included throughout the workflow and processed in parallel with clinical samples. Purified DNA products were pooled to generate the final MiSeq library, which was submitted to a local commercial sequencing facility. Library was sequenced on an Illumina platform (e.g., MiSeq) using paired-end reads (2 × 250 bp). A target sequencing depth of ~30,000–50,000 reads per sample was applied to ensure adequate coverage of microbial diversity.

### 2.6. Bioinformatics and data analysis

Raw sequencing data generated by the Illumina MiSeq platform consisted of paired-end FASTQ files (R1 and R2) for each sample. Initial quality control, sequence processing, and taxonomic assignment were performed using Illumina’s proprietary bioinformatics pipeline. Briefly, paired-end reads were quality-filtered and aligned against a curated reference database using a BLAST-based approach, in which homologous sequences were identified through local alignments of short matching regions between query sequences and reference 16S rRNA gene sequences. Sequences obtained from negative control samples were also analyzed to assess potential background contamination and to support interpretation of low-abundance taxa in clinical samples. Taxonomic classification of bacterial taxa was derived from the resulting alignment output and summarized in a microbiome analysis report.

## 3. Results

### 3.1. Classical identification culture method

In this study, 30 urine samples were analyzed using culture methods. Of these, 15 samples exhibited no significant bacterial growth, and 4 samples showed negative growth. The most frequently identified bacterial species were *Escherichia coli*, followed by *Klebsiella spp.*, *Lactobacillus spp.*, *Enterococcus spp.*, *Group B Streptococcus*, *Acinetobacter spp.*, and *Proteus mirabilis*. While species identification was made in some cases, these were primarily based on known pathogenic genera commonly found in human urine samples.

For cerebrospinal fluid (CSF) samples, bacterial growth was observed in 4 of the positive samples, where the following species were identified: *Coagulase-negative Staphylococcus* (CoNS), *Enterobacter spp.*, and *Methicillin-resistant Staphylococcus aureus* (MRSA). The remaining samples showed no bacterial growth by culture.

### 3.2. NGS microbiome analysis: Urine and CSF samples

Next-generation sequencing (NGS) was employed to further characterize the microbiome present in the urine and CSF samples. The analysis revealed detailed species and genus-level identifications, with samples categorized into groups based on bacterial growth: urine samples with positive growth, urine samples with insignificant growth, urine samples with no growth, CSF samples with positive growth, and CSF samples with no growth.

**Fig 1:**
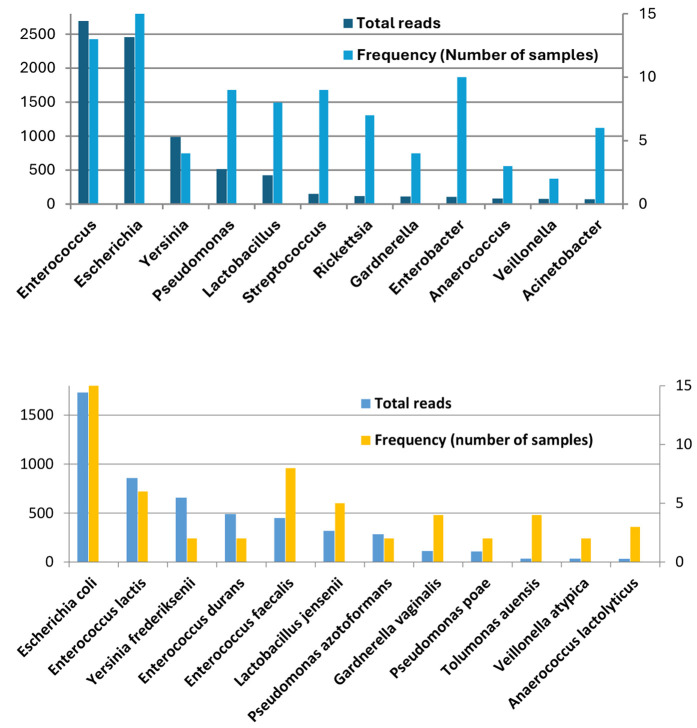
Abundance and Frequency of Bacterial Genera (A) and Species (B) in Urine Samples with Positive Growth. Panel (A) displays the most abundant bacterial genera detected by NGS analysis in urine samples with positive bacterial growth from culture methods. Panel (B) shows the species-level distribution of the most frequently occurring bacteria in the same samples.

**Fig 2:**
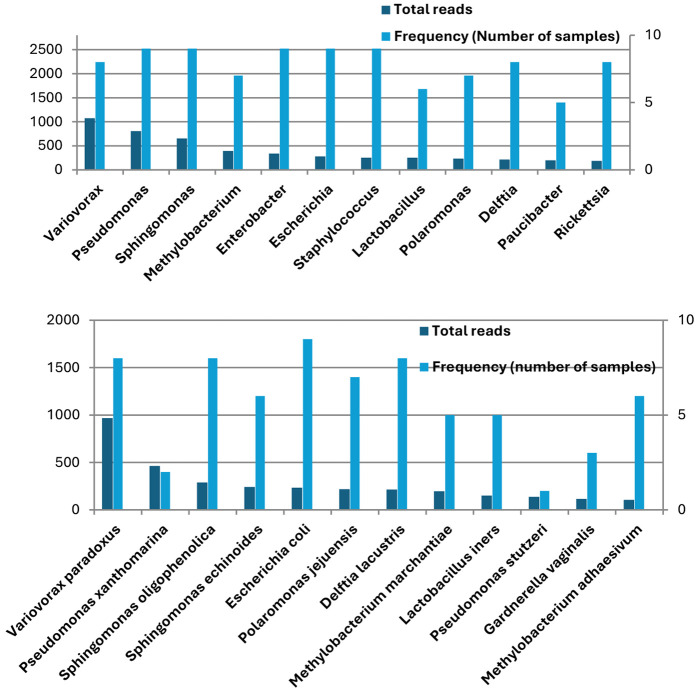
Abundance and Frequency of Bacterial Genera and Species in Urine Samples with Insignificant Growth. The upper histogram shows the most abundant bacterial genera identified by NGS analysis in urine samples with insignificant bacterial growth, as determined by classical culture methods. The lower histogram depicts the species-level distribution of the most frequently occurring bacteria in the same samples.

**Fig 3:**
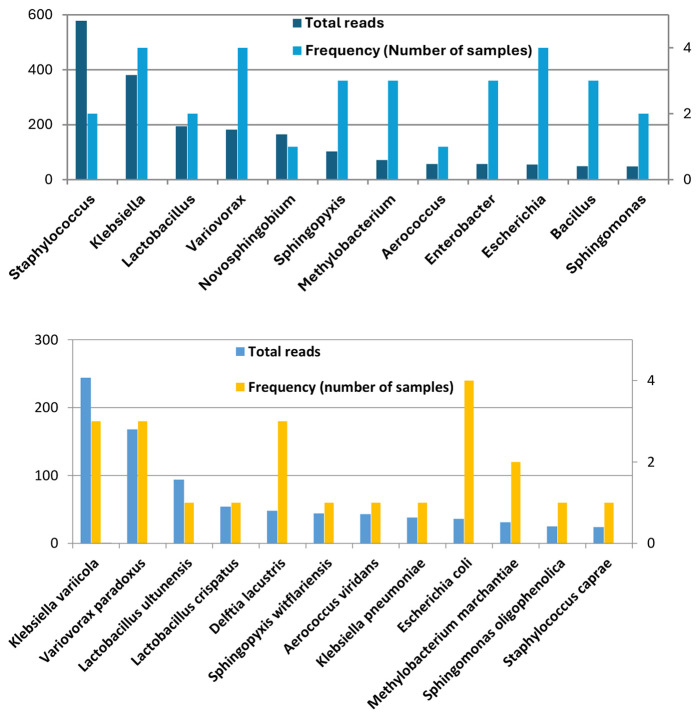
Abundance and Frequency of Bacterial Genera and Species in Urine Samples with Negative Growth. The upper histogram displays the most abundant bacterial genera identified by NGS analysis in urine samples showing no bacterial growth by classical culture methods. The lower histogram shows the species-level distribution of the most frequent bacteria in the same samples.

**Fig 4:**
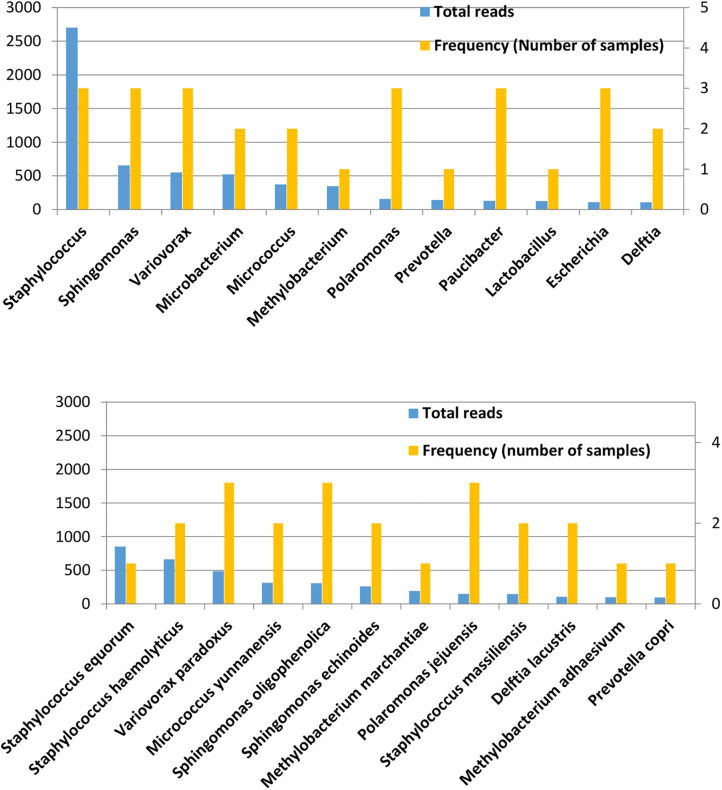
Abundance and Frequency of Bacterial Species in CSF Samples with Positive Growth. The upper histogram shows the most abundant bacterial species detected by NGS analysis in CSF samples with positive bacterial growth according to classical culture methods. The lower histogram displays the species most frequently encountered in the same samples.

**Fig 5:**
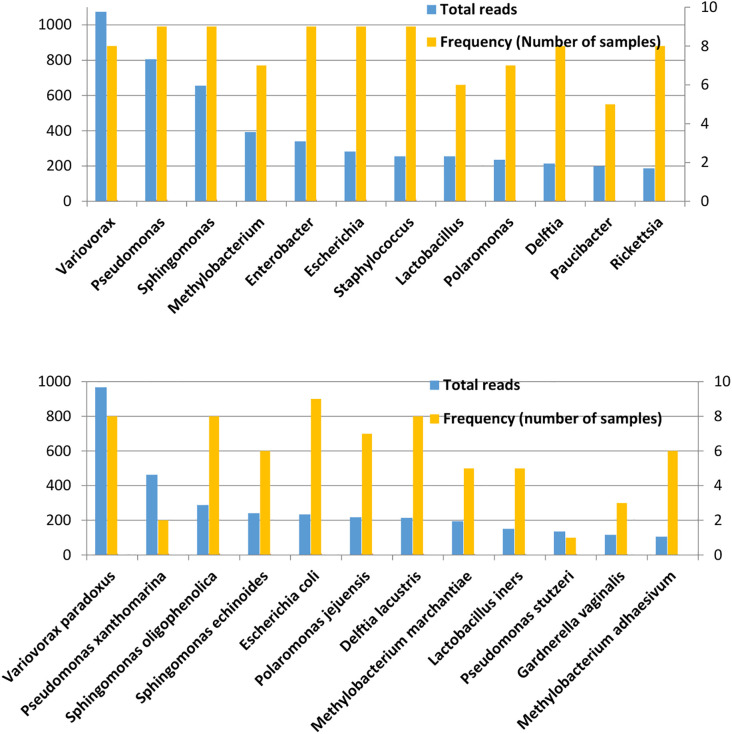
Abundance and Frequency of Bacterial Genera in CSF Samples with Negative Growth. The upper histogram displays the most abundant bacterial genera detected by NGS analysis in CSF samples with no bacterial growth by classical culture methods. The lower histogram shows the bacterial genera most frequently found in the same samples.

FASTQ files were processed into FASTA format to determine the total number of amplicon reads per sample group. Histograms (Figs 1–5) illustrate the bacterial genera, species, and read frequencies detected in each sample category. Detailed results are provided in S1–S10 Tables (Supplementary Information).

### 3.3. Urine samples with significant bacterial growth (≥10^5^ CFU/mL)

The NGS analysis of urine samples with significant bacterial growth (≥10^5^ CFU/mL) revealed *Enterococcus* as the most abundant genus, with 2455 reads across 13 of the 30 positive samples. This genus is a common nosocomial pathogen associated with urinary tract and intra-abdominal infections. *Escherichia* was the second most abundant genus, with a higher frequency of 15 out of 30 samples. Other frequently detected genera included *Klebsiella*, which is a known uropathogen, detected in 6 out of 30 samples, with 30 amplicon reads (Fig 1).

At the species level, *Escherichia coli* was the most prevalent, with 1732 reads, detected in 15 out of 30 samples. *Enterococcus faecalis* was also prominent, present in 8 of 30 positive samples with 450 reads. *Gardnerella vaginalis*, with 113 reads in 4 samples, is typically associated with vaginitis and may have been detected due to contamination or poor hygiene.

### 3.4. Urine samples with non-significant bacterial growth (<10³ CFU/mL)

In urine samples with **non-significant bacterial growth (<10³ CFU/mL)**, the NGS analysis still detected multiple bacterial taxa. The most abundant genera were *Escherichia* (2626 reads) and *Enterococcus* (2150 reads) as the most abundant genera. Other notable genera included *Pseudomonas* (1178 reads) and *Lactobacillus* (S3 Table). *Escherichia coli* was found in 9 out of 15 samples in this group, as shown in Fig 2 and S4 Table.

### 3.5. Urine samples with no growth by culture (0 CFU/mL)

For urine samples with no **detectable** bacterial growth **by conventional culture (0 CFU/mL)**, NGS analysis detected several genera: *Staphylococcus* (578 reads), *Klebsiella* (381 reads), and *Lactobacillus* (194 reads) (S5 Table). The species *Klebsiella variicola*, a facultative anaerobic Gram-negative bacillus, was identified in 3 out of 5 negative samples, while *Escherichia coli* was present in 4 out of 5 negative samples (Fig 3, S6 Table).

### 3.6. CSF samples with positive growth

In CSF samples, bacterial growth was observed in 4 of the 10 samples. The predominant genus identified was *Staphylococcus* (2702 reads), which is commonly associated with meningitis, particularly following surgical procedures or bloodstream infections. *Staphylococcus haemolyticus*, with 664 reads, was the most abundant species in these samples, detected in 2 of 4 positive samples (Fig 4, S7, S8 Table).

### 3.7. CSF samples with no growth

Among the CSF samples showing no bacterial growth by classical culture, the NGS analysis identified several genera with varying abundance: *Pseudomonas* (805 reads), *Enterobacter* (340 reads), *Escherichia* (282 reads), *Staphylococcus* (255 reads), and *Rickettsia* (187 reads). The most frequent genera across the negative samples were *Enterobacter*, *Pseudomonas*, *Escherichia*, and *Staphylococcus* (Fig 5, S9 Table). The species Escherichia coli was the most common, with 234 reads detected in 1 out of 10 negative samples (S10 Table).

## 4. Discussion

Culture-based methods are widely regarded as the gold standard for bacterial diagnosis [35]. However, these methods present significant limitations, particularly for slow-growing or fastidious pathogens, resulting in prolonged turnaround times and reduced diagnostic sensitivity. Timely pathogen identification is critical for minimizing patient morbidity and mortality, limiting inappropriate use of broad-spectrum antibiotics, and reducing hospital-acquired infection rates [36].

Molecular identification using nucleotide sequences of the 16S rRNA gene has emerged as an effective alternative to culture-based diagnostics. This conserved gene is present in approximately 96% of bacterial genera and 87.5% of bacterial species, allowing broad taxonomic coverage. Sequencing-based approaches reduce culture-associated biases, particularly in cases with negative or inconclusive culture results [37]. Illumina MiSeq NGS has been widely applied in metagenomic studies to analyze complex microbial populations from a single clinical specimen. Universal primers targeting the bacterial 16S rRNA gene enable simultaneous amplification of multiple organisms [1]. In contrast to classical sequencing, NGS provides both qualitative and quantitative data for each amplicon while eliminating labor-intensive cloning steps. The resulting abundance of sequence reads reflects the relative prevalence of microorganisms within the original sample [38]. The principal advantages of NGS therefore include its high sensitivity, precision, and scalability [39, 40]. It should be noted that modifications to the PCR protocol, including an increased number of amplification cycles, may introduce amplification bias or background signal, particularly in low-biomass samples; however, these adjustments were necessary to enhance detection sensitivity.

Recent advances in nanopore-based sequencing technologies, particularly those developed by Oxford Nanopore Technologies, have demonstrated significant potential for real-time pathogen detection in clinical microbiology. Unlike Illumina-based short-read sequencing, nanopore sequencing enables long-read, single-molecule analysis and real-time data acquisition, allowing pathogen identification within hours rather than days [41]. In addition, nanopore sequencing offers advantages such as the detection of viable but non-culturable organisms and simultaneous identification of antimicrobial resistance genes. However, despite these advantages, limitations remain, including relatively higher raw read error rates and variability in sequencing accuracy compared to established short-read platforms. Therefore, while nanopore sequencing represents a promising and rapidly evolving tool, Illumina-based 16S rRNA sequencing remains a robust and widely validated approach for microbiome profiling. These technologies should be viewed as complementary, with nanopore sequencing offering speed and flexibility, and short-read NGS providing high accuracy and established analytical pipelines [42, 43].

Despite these advantages, NGS implementation in clinical settings presents challenges. Although highly sensitive, NGS requires substantial infrastructure and specialized expertise to manage and interpret large datasets. Furthermore, the cost remains prohibitive for routine diagnostics, and incomplete reference databases particularly for fungal pathogens—may lead to ambiguous or misleading results. These limitations highlight the importance of evaluating NGS as a complementary rather than replacement diagnostic tool [44].

In the present study, bacterial species were characterized in urine and cerebrospinal fluid (CSF) samples obtained from patients with suspected infections. Infected urine samples commonly contained Escherichia coli, Staphylococcus saprophyticus, Klebsiella pneumoniae, Staphylococcus aureus, Enterococcus faecalis, Proteus mirabilis, Group B Streptococcus, and Pseudomonas aeruginosa [24]. Samples were analyzed using both culture-dependent and culture-independent methods (PCR and NGS). While both approaches identified the most of the major bacterial pathogens, NGS additionally detected bacterial genera not recovered by cultivation. S1 and S2 Tables summarize the read counts and frequencies of bacterial pathogens in urine samples. The Enterococcus genus yielded approximately 2455 reads in 13 out of 30 positive urine samples by NGS, compared with detection in 7 out of 30 samples by culture. Escherichia coli was the most abundant species, with 1732 reads recorded in positive samples. Traditional culture identified Escherichia coli, Klebsiella spp., Lactobacillus spp., Enterococcus spp., Group B Streptococcus, Acinetobacter spp., and Proteus mirabilis, whereas NGS additionally detected Pseudomonas, Rickettsia, and Gardnerella. Gardnerella was identified exclusively by NGS, potentially due to its Gram-variable cell wall and poor culturability. Conversely, Klebsiella was detected by culture but not by NGS.

Urine samples classified as having insignificant growth by culture were further evaluated (S3 and S4 Tables). NGS detected multiple bacterial genera not identified by cultivation, reflecting its ability to detect low-abundance organisms. Pseudomonas was the most abundant genus (750 reads) and the most frequently detected, occurring in 10 out of 15 samples, while Escherichia coli was the most abundant individual species. Many detected organisms were facultative, obligate anaerobic, or fastidious bacteria that are difficult to cultivate using standard methods [45, 46]

In the five urine samples classified as culture-negative, NGS detected Streptococcus, Enterobacter, Escherichia, Lactobacillus, and Klebsiella. Staphylococcus was the most abundant genus (578 reads), detected in 2 out of 5 samples, while Klebsiella exhibited approximately 380 reads and was detected in 4 out of 5 samples. Detailed results are presented in Tables S5 and S6. These findings further support evidence that the bladder is not sterile and that non-cultivable bacteria are frequently present in urine [25, 26].

Despite an overall overlap in the detection of urinary tract pathogens such as *Escherichia coli* and *Enterococcus spp.*, discrepancies were observed between culture and NGS results. In some cases, organisms detected by culture were not identified by NGS, and conversely, NGS detected bacterial DNA in culture-negative samples. These differences may reflect methodological factors, including variations in sensitivity, detection of non-viable organisms by NGS, PCR bias, and potential contamination.

CSF is normally sterile but may become infected by a wide range of bacterial pathogens, potentially leading to meningitis and central nervous system involvement. Common causative organisms include Streptococcus agalactiae, Escherichia coli, Neisseria meningitidis, Streptococcus pneumoniae, Haemophilus influenzae, Listeria monocytogenes, Mycobacterium tuberculosis, Treponema pallidum, Leptospira spp., and Borrelia burgdorferi [47]. Rapid diagnosis is essential to prevent mortality and severe neurological sequelae, as viruses and bacteria are the predominant etiological agents of meningitis [48].

Culture-based analysis of four positive CSF samples identified coagulase-negative Staphylococcus spp. (CoNS), Enterobacter spp., and methicillin-resistant Staphylococcus aureus (MRSA). Failure to detect pathogens in other samples may be attributed to antibiotic exposure or infection stage. NGS detected Staphylococcus in all positive samples, with the highest read count (2702) and a frequency of 3 out of 4 samples. Staphylococcus haemolyticus, a CoNS species associated with nosocomial neurosurgical infections, was the most abundant species [49]. Detailed results are presented in S7 and S8 Tables.

The relatively small number of cerebrospinal fluid samples and limited clinical characterization of some specimens represent important limitations of this study. This constraint is partly influenced by the local epidemiological context, as widespread vaccination programs in the West Bank have reduced the incidence of bacterial infections, particularly those affecting cerebrospinal fluid, thereby limiting sample availability. Accordingly, the results should be interpreted with caution, and further studies with larger, well-characterized cohorts are needed.

Growing evidence supports the use of 16S rRNA gene sequencing as a rapid and accurate diagnostic tool for CSF analysis [50]. In ten CSF samples classified as culture-negative, NGS detected Pseudomonas, Escherichia, Staphylococcus, and Enterobacter. Pseudomonas was the most abundant genus (805 reads), detected in 9 out of 10 samples. Escherichia coli was the most abundant species, with 234 reads and a frequency of 9 out of 10 samples. These findings are summarized in S9 and S10 Tables.

NGS-based diagnostics are increasingly implemented in clinical microbiology laboratories. Their ability to directly detect known and unknown pathogens in clinical specimens provides a substantial advantage over conventional culture-based methods. Current evidence suggests that NGS should complement rather than replace traditional diagnostics [45, 51] While cost, turnaround time, and technical complexity remain barriers, continued technological advances are expected to expand routine clinical applications [45]. The use of 16S rRNA NGS is particularly valuable for detecting fastidious, slow-growing, or non-viable bacteria that challenge standard cultivation techniques, although interpretation requires specialized bioinformatics expertise and validated workflows [37].

Overall, NGS confirmed all positive culture-based identifications in both urine and CSF samples and demonstrated superior sensitivity by detecting slow-growing, fastidious, and low-abundance bacteria in samples classified as insignificant or negative by culture. These findings support the role of NGS as a high-sensitivity complementary tool in clinical microbiology diagnostics. NGS showed a high but incomplete concordance with culture-based identification, confirming most clinically significant isolates while also demonstrating discrepancies in a limited number of cases.

## Supporting information

S1 Table The most common microorganisms obtained by NGS DNA sequence analysis from urine-positive samples, classified based on genus. (DOCX)

S2 Table The most common microorganisms obtained by NGS DNA sequence analysis from urine-positive samples, classified based on species. (DOCX)

S3 Table The most common microorganisms obtained by NGS DNA sequence analysis from urine samples that showed no significant bacterial growth, classified based on genus. (DOCX)

S4 Table The most common microorganisms obtained by NGS DNA sequence analysis from urine samples that showed no significant bacterial growth, classified based on species. (DOCX)

S5 Table The most common microorganisms obtained by NGS DNA sequence analysis from urine samples that showed no bacterial growth, classified based on genus. (DOCX)

S6 Table The most common microorganisms obtained by NGS DNA sequence analysis from urine samples that showed no bacterial growth, classified based on species. (DOCX)

S7 Table The most common microorganisms obtained by NGS DNA sequence analysis from CSF samples that showed positive bacterial growth, classified based on genus. (DOCX)

S8 Table The most common microorganisms obtained by NGS DNA sequence analysis from CSF samples that showed positive bacterial growth, classified based on species. (DOCX)

S9 Table The most common microorganisms obtained by NGS DNA sequence analysis from CSF samples that showed negative bacterial growth, classified based on genus. (DOCX)

S10 Table The most common microorganisms obtained by NGS DNA sequence analysis from CSF samples that showed negative bacterial growth, classified based on species. (DOCX)
