# Supplementary material for: A comparison between bacterial cultivation and 16S rRNA next generation sequencing approaches for analysis of bacteria in urine and cerebrospinal fluid samples
Source: PLoS One. 2026 Jun 25;21(6):e0350939. doi: 10.1371/journal.pone.0350939 (PMC13298949; doi:10.1371/journal.pone.0350939)
Supplement: S1 Table — (DOCX) [file pone.0350939.s001.docx]

**S1 Table:** The most common microorganisms obtained by NGS DNA sequence analysis from urine-positive samples , classified based on genus.

| **Bacterial genus** | **Total reads** | **Frequency (Sample Number)** |
| --- | --- | --- |
| *Enterococcus* | 2693 | 13 |
| *Escherichia* | 2455 | 15 |
| *Yersinia* | 987 | 4 |
| *Pseudomonas* | 515 | 9 |
| *Lactobacillus* | 423 | 8 |
| *Streptococcus* | 150 | 9 |
| *Rickettsia* | 118 | 7 |
| *Gardnerella* | 113 | 4 |
| *Enterobacter* | 107 | 10 |
| *Anaerococcus* | 83 | 3 |
| *Veillonella* | 76 | 2 |
| *Acinetobacter* | 69 | 6 |
| *Peptoniphilus* | 56 | 2 |
| *Ralstonia* | 52 | 4 |
| *Varibaculum* | 45 | 1 |
| *Nevskia* | 41 | 6 |
| *Prevotella* | 41 | 5 |
| *Corynebacterium* | 40 | 6 |
| *Tolumonas* | 34 | 4 |
| *Klebsiella* | 30 | 6 |
| *Gemella* | 28 | 1 |
| *Rothia* | 27 | 1 |
| *Providencia* | 25 | 5 |
| *Agrobacterium* | 24 | 4 |
| *Staphylococcus* | 21 | 6 |
| *Stenotrophomonas* | 21 | 5 |
| *Alkaliphilus* | 19 | 4 |
| *Micrococcus* | 19 | 2 |
| *Porphyromonas* | 19 | 1 |
| *Limnobacter* | 18 | 6 |
| *Variovorax* | 18 | 6 |
| *Bacillus* | 18 | 5 |
| *Actinomyces* | 18 | 4 |
| *Methylobacterium* | 17 | 3 |
| *Negativicoccus* | 15 | 1 |
| *Peptostreptococcus* | 14 | 2 |
| *Leptotrichia* | 13 | 1 |
| *Thiomonas* | 11 | 4 |
| *Swaminathania* | 11 | 2 |
| *Novosphingobium* | 11 | 2 |
| *Mobiluncus* | 11 | 2 |
