# Supplementary material for: A comparison between bacterial cultivation and 16S rRNA next generation sequencing approaches for analysis of bacteria in urine and cerebrospinal fluid samples
Source: PLoS One. 2026 Jun 25;21(6):e0350939. doi: 10.1371/journal.pone.0350939 (PMC13298949; doi:10.1371/journal.pone.0350939)
Supplement: S2 Table — (DOCX) [file pone.0350939.s002.docx]

**S2 Table:** The most common microorganisms obtained by NGS DNA sequence analysis from urine-positive samples, classified based on species.

| **Bacterial species** | **Total reads** | **Frequency (Sample Number)** |
| --- | --- | --- |
| *Escherichia coli* | 1732 | 15 |
| *Enterococcus lactis* | 857 | 6 |
| *Yersinia frederiksenii* | 657 | 2 |
| *Enterococcus durans* | 489 | 2 |
| *Enterococcus faecalis* | 450 | 8 |
| *Lactobacillus jensenii* | 318 | 5 |
| *Pseudomonas azotoformans* | 284 | 2 |
| *Gardnerella vaginalis* | 113 | 4 |
| *Pseudomonas poae* | 108 | 2 |
| *Tolumonas auensis* | 34 | 4 |
| *Veillonella atypica* | 34 | 2 |
| *Anaerococcus lactolyticus* | 33 | 3 |
| *Enterobacter aceae* | 29 | 7 |
| *Acinetobacter tjernbergiae* | 28 | 2 |
| *Lactobacillus iners* | 28 | 1 |
| *Rothia mucilaginosa* | 24 | 1 |
| *Nevskia ramosa* | 22 | 6 |
| *Enterobacter nickellidurans* | 22 | 2 |
| *Streptococcus parasanguinis* | 22 | 1 |
| *Lactobacillus gigeriorum* | 21 | 1 |
| *Veillonella dispar* | 20 | 1 |
| *Streptococcus anginosus* | 20 | 1 |
| *Lactobacillus taiwanensis* | 17 | 3 |
| *Micrococcus yunnanensis* | 17 | 2 |
| *Klebsiella oxytoca* | 17 | 1 |
| *Variovorax paradoxus* | 15 | 5 |
| *Providencia rettgeri* | 14 | 4 |
| *Streptococcus tigurinus* | 14 | 3 |
| *Peptoniphilus gorbachii* | 14 | 1 |
| *Peptostreptococcus anaerobius* | 13 | 2 |
| *Stenotrophomonas pavanii* | 12 | 5 |
| *Streptococcus pseudopneumoniae* | 12 | 3 |
| *Ralstonia detusculanense* | 12 | 3 |
| *Prevotella melaninogenica* | 12 | 1 |
| *Streptococcus bovis* | 12 | 1 |
| *Anaerococcus tetradius* | 12 | 1 |
| *Pseudomonas chloritidismutans* | 12 | 1 |
| *Thiomonas thermosulfata* | 11 | 4 |
| *Enterobacter ludwigii* | 11 | 3 |
| *Yersinia massiliensis* | 11 | 1 |
