# Supplementary material for: A comparison between bacterial cultivation and 16S rRNA next generation sequencing approaches for analysis of bacteria in urine and cerebrospinal fluid samples
Source: PLoS One. 2026 Jun 25;21(6):e0350939. doi: 10.1371/journal.pone.0350939 (PMC13298949; doi:10.1371/journal.pone.0350939)
Supplement: S3 Table — (DOCX) [file pone.0350939.s003.docx]

**S3 Table:** The most common microorganisms obtained by NGS DNA sequence analysis from urine samples that showed no significant bacterial growth, classified based on genus.

| **Bacterial genus** | **Total reads** | **Frequency (Sample Number)** |
| --- | --- | --- |
| *Escherichia* | 2626 | 9 |
| *Enterococcus* | 2150 | 6 |
| *Pseudomonas* | 1178 | 8 |
| *Lactobacillus* | 966 | 6 |
| *Lactococcus* | 822 | 2 |
| *Neisseria* | 636 | 2 |
| *Gluconacetobacter* | 606 | 1 |
| *Gardnerella* | 317 | 3 |
| *Yersinia* | 245 | 4 |
| *Facklamia* | 187 | 2 |
| *Corynebacterium* | 149 | 9 |
| *Pediococcus* | 127 | 4 |
| *Megasphaera* | 125 | 1 |
| *Prevotella* | 93 | 7 |
| *Enterobacter* | 68 | 8 |
| *Peptoniphilus* | 66 | 6 |
| *Atopobium* | 58 | 2 |
| *Tolumonas* | 56 | 3 |
| *Proteus* | 55 | 2 |
| *Psychrobacter* | 51 | 2 |
| *Variovorax* | 46 | 5 |
| *Clostridium* | 44 | 3 |
| *Aerococcus* | 41 | 2 |
| *Staphylococcus* | 32 | 6 |
| *Stenotrophomonas* | 27 | 5 |
| *Helcococcus* | 27 | 1 |
| *Alkaliphilus* | 25 | 2 |
| *Streptococcus* | 24 | 3 |
| *Acinetobacter* | 19 | 4 |
| *Nevskia* | 17 | 4 |
| *Rickettsia* | 15 | 7 |
| *Bifidobacterium* | 14 | 4 |
| *Alloscardovia* | 14 | 3 |
| *Providencia* | 13 | 3 |
| *Vagococcus* | 12 | 2 |
| *Paucibacter* | 11 | 3 |
| *Methylobacterium* | 11 | 2 |
| *Anaerococcus* | 11 | 1 |
