# Supplementary material for: A comparison between bacterial cultivation and 16S rRNA next generation sequencing approaches for analysis of bacteria in urine and cerebrospinal fluid samples
Source: PLoS One. 2026 Jun 25;21(6):e0350939. doi: 10.1371/journal.pone.0350939 (PMC13298949; doi:10.1371/journal.pone.0350939)
Supplement: S4 Table — (DOCX) [file pone.0350939.s004.docx]

**S4 Table:** The most common microorganisms obtained by NGS DNA sequence analysis from urine samples that showed no significant bacterial growth, classified based on species.

| **Bacterial species** | **Total reads** | **Frequency (Sample Number)** |
| --- | --- | --- |
| *Escherichia coli* | 1991 | 9 |
| *Enterococcus faecalis* | 843 | 3 |
| *Enterococcus faecalis* | 843 | 3 |
| *Lactococcus raffinolactis* | 673 | 1 |
| *Lactobacillus iners* | 540 | 4 |
| *Pseudomonas lundensis* | 522 | 3 |
| *Pseudomonas fragi* | 408 | 2 |
| *Gardnerella vaginalis* | 314 | 3 |
| *Neisseria mucosa* | 303 | 2 |
| *Enterococcus lactis* | 226 | 3 |
| *Yersinia frederiksenii* | 129 | 3 |
| *Neisseria lactamica* | 121 | 1 |
| *Lactobacillus ultunensis* | 97 | 3 |
| *Lactobacillus taiwanensis* | 84 | 5 |
| *Pediococcus stilesii* | 84 | 4 |
| *Lactobacillus crispatus* | 81 | 2 |
| *Pseudomonas azotoformans* | 74 | 1 |
| *Enterococcus durans* | 71 | 1 |
| *Prevotella timonensis* | 61 | 6 |
| *Tolumonas auensis* | 56 | 3 |
| *Lactobacillus jensenii* | 52 | 2 |
| *Yersinia kristensenii* | 47 | 1 |
| *Variovorax paradoxus* | 42 | 4 |
| *Proteus penneri* | 30 | 2 |
| *Corynebacterium flavescens* | 30 | 1 |
| *Aerococcus christensenii* | 29 | 1 |
| *Yersinia massiliensis* | 29 | 1 |
| *Pediococcus cellicola* | 27 | 4 |
| *Psychrobacter pulmonis* | 27 | 2 |
| *Pseudomonas tremae* | 24 | 1 |
| *Prevotella bivia* | 22 | 3 |
| *Enterobacter aceae* | 21 | 5 |
| *Facklamia tabacinasalis* | 21 | 1 |
| *Helcococcus sueciensis* | 21 | 1 |
| *Peptoniphilus asaccharolyticus* | 19 | 4 |
| *Corynebacterium simulans* | 17 | 1 |
| *Enterobacter hormaechei* | 16 | 3 |
| *Alkaliphilus peptidifermentans* | 16 | 2 |
| *Escherichia coli* | 13 | 3 |
| *Peptoniphilus gorbachii* | 12 | 4 |
| *Vagococcus teuberi* | 12 | 2 |
| *Lactobacillus acidophilus* | 11 | 2 |
