# Supplementary material for: A comparison between bacterial cultivation and 16S rRNA next generation sequencing approaches for analysis of bacteria in urine and cerebrospinal fluid samples
Source: PLoS One. 2026 Jun 25;21(6):e0350939. doi: 10.1371/journal.pone.0350939 (PMC13298949; doi:10.1371/journal.pone.0350939)
Supplement: S5 Table — (DOCX) [file pone.0350939.s005.docx]

**S5 Table:** The most common microorganisms obtained by NGS DNA sequence analysis from urine samples that showed no bacterial growth, classified based on genus.

| **Bacterial genus** | **Total reads** | **Frequency (Sample Number)** |
| --- | --- | --- |
| *Klebsiella* | 381 | 4 |
| *Variovorax* | 182 | 4 |
| *Escherichia* | 55 | 4 |
| *Providencia* | 35 | 4 |
| *Corynebacterium* | 19 | 4 |
| *Microvirus* | 6 | 4 |
| *Sphingopyxis* | 103 | 3 |
| *Methylobacterium* | 71 | 3 |
| *Enterobacter* | 57 | 3 |
| *Bacillus* | 49 | 3 |
| *Delftia* | 48 | 3 |
| *Pseudomonas* | 46 | 3 |
| *Enterococcus* | 44 | 3 |
| *Paucibacter* | 38 | 3 |
| *Serratia* | 30 | 3 |
| *Prevotella* | 23 | 3 |
| *Limnobacter* | 20 | 3 |
| *Nevskia* | 16 | 3 |
| *Heliorestis* | 4 | 3 |
| *Staphylococcus* | 578 | 2 |
| *Lactobacillus* | 194 | 2 |
| *Sphingomonas* | 48 | 2 |
| *Rickettsia* | 34 | 2 |
| *Acinetobacter* | 27 | 2 |
| *Streptococcus* | 19 | 2 |
| *Faecalibacterium* | 18 | 2 |
| *Azohydromonas* | 18 | 2 |
| *Yersinia* | 17 | 2 |
| *Polaromonas* | 16 | 2 |
| *Tolumonas* | 13 | 2 |
| *Burkholderia* | 11 | 2 |
