# Supplementary material for: A comparison between bacterial cultivation and 16S rRNA next generation sequencing approaches for analysis of bacteria in urine and cerebrospinal fluid samples
Source: PLoS One. 2026 Jun 25;21(6):e0350939. doi: 10.1371/journal.pone.0350939 (PMC13298949; doi:10.1371/journal.pone.0350939)
Supplement: S6 Table — (DOCX) [file pone.0350939.s006.docx]

**S6 Table:** The most common microorganisms obtained by NGS DNA sequence analysis from urine samples that showed no bacterial growth, classified based on species.

| **Bacterial species** | **Total reads** | **Frequency (Sample Number)** |
| --- | --- | --- |
| *Klebsiella variicola* | 244 | 3 |
| *Variovorax paradoxus* | 168 | 3 |
| *Lactobacillus ultunensis* | 94 | 1 |
| *Lactobacillus crispatus* | 54 | 1 |
| *Delftia lacustris* | 48 | 3 |
| *Sphingopyxis witflariensis* | 44 | 1 |
| *Aerococcus viridans* | 43 | 1 |
| *Klebsiella pneumoniae* | 38 | 1 |
| *Escherichia coli* | 36 | 4 |
| *Methylobacterium marchantiae* | 31 | 2 |
| *Sphingomonas oligophenolica* | 25 | 1 |
| *Staphylococcus caprae* | 24 | 1 |
| *Novosphingobium aromaticivorans* | 23 | 1 |
| *Providencia rettgeri* | 17 | 4 |
| *Klebsiella granulomatis* | 17 | 2 |
| *Enterobacter amnigenus* | 16 | 3 |
| *Methylobacterium goesingense* | 16 | 2 |
| *Enterococcus faecalis* | 16 | 2 |
| *Providencia sneebia* | 16 | 2 |
| *Variovorax boronicumulans* | 14 | 4 |
| *Nevskia ramosa* | 14 | 3 |
| *Yersinia frederiksenii* | 14 | 2 |
| *Polaromonas jejuensis* | 14 | 2 |
| *Sphingomonas echinoides* | 14 | 1 |
| *Tolumonas auensis* | 13 | 2 |
| *Methylobacterium adhaesivum* | 12 | 2 |
| *Ralstonia pickettii* | 12 | 1 |
| *Sphingopyxis chilensis* | 11 | 2 |
| *Enterobacter nickellidurans* | 11 | 2 |
| *Bacillus litoralis* | 11 | 1 |
