# Supplementary material for: A comparison between bacterial cultivation and 16S rRNA next generation sequencing approaches for analysis of bacteria in urine and cerebrospinal fluid samples
Source: PLoS One. 2026 Jun 25;21(6):e0350939. doi: 10.1371/journal.pone.0350939 (PMC13298949; doi:10.1371/journal.pone.0350939)
Supplement: S7 Table — (DOCX) [file pone.0350939.s007.docx]

**S7 Table:** The most common microorganisms obtained by NGS DNA sequence analysis from CSF samples that showed positive bacterial growth, classified based on genus.

| **Bacterial genus** | **Total reads** | **Frequency (Sample Number)** |
| --- | --- | --- |
| *Staphylococcus* | 2702 | 3 |
| *Sphingomonas* | 655 | 3 |
| *Variovorax* | 552 | 3 |
| *Microbacterium* | 524 | 2 |
| *Micrococcus* | 373 | 2 |
| *Methylobacterium* | 346 | 1 |
| *Polaromonas* | 158 | 3 |
| *Prevotella* | 142 | 1 |
| *Paucibacter* | 131 | 3 |
| *Lactobacillus* | 127 | 1 |
| *Escherichia* | 111 | 3 |
| *Delftia* | 107 | 2 |
| *Rathayibacter* | 105 | 1 |
| *Oerskovia* | 97 | 1 |
| *Faecalibacterium* | 85 | 1 |
| *Streptococcus* | 75 | 2 |
| *Citricoccus* | 73 | 1 |
| *Ureaplasma* | 71 | 1 |
| *Pseudomonas* | 53 | 3 |
| *Bacillus* | 52 | 2 |
| *Ralstonia* | 44 | 1 |
| *Serratia* | 43 | 2 |
| *Acinetobacter* | 43 | 2 |
| *Sediminibacterium* | 40 | 1 |
| *Bacteroides* | 39 | 1 |
| *Oscillospira* | 38 | 1 |
| *Kocuria* | 37 | 2 |
| *Rhodococcus* | 35 | 1 |
| *Succinivibrio* | 27 | 1 |
| *Pediococcus* | 23 | 1 |
| *Arthrobacter* | 22 | 1 |
| *Corynebacterium* | 19 | 2 |
| *Veillonella* | 17 | 1 |
| *Agrobacterium* | 16 | 2 |
| *Blautia* | 15 | 2 |
| *Porphyromonas* | 14 | 1 |
| *Clostridium* | 13 | 2 |
| *Paracoccus* | 13 | 1 |
| *Rickettsia* | 12 | 3 |
| *Providencia* | 12 | 2 |
| *Parabacteroides* | 11 | 1 |
| *Ruminococcus* | 11 | 1 |
