# Supplementary material for: A comparison between bacterial cultivation and 16S rRNA next generation sequencing approaches for analysis of bacteria in urine and cerebrospinal fluid samples
Source: PLoS One. 2026 Jun 25;21(6):e0350939. doi: 10.1371/journal.pone.0350939 (PMC13298949; doi:10.1371/journal.pone.0350939)
Supplement: S8 Table — (DOCX) [file pone.0350939.s008.docx]

**S8 Table:** The most common microorganisms obtained by NGS DNA sequence analysis from CSF samples that showed positive bacterial growth, classified based on species.

| **Bacterial species** | **Total reads** | **Frequency (Sample Number)** |
| --- | --- | --- |
| *Variovorax paradoxus* | 486 | 3 |
| *Sphingomonas oligophenolica* | 307 | 3 |
| *Polaromonas jejuensis* | 150 | 3 |
| *Staphylococcus haemolyticus* | 664 | 2 |
| *Micrococcus yunnanensis* | 312 | 2 |
| *Sphingomonas echinoides* | 261 | 2 |
| *Staphylococcus massiliensis* | 146 | 2 |
| *Delftia lacustris* | 107 | 2 |
| *Escherichia coli* | 96 | 2 |
| *Microbacterium maritypicum* | 92 | 2 |
| *Serratia entomophila* | 43 | 2 |
| *Microbacterium marinilacus* | 37 | 2 |
| *Microbacterium hydrocarbonoxydans* | 31 | 2 |
| *Kocuria gwangalliensis* | 26 | 2 |
| *Staphylococcus caprae* | 25 | 2 |
| *Acinetobacter tjernbergiae* | 12 | 2 |
