# Supplementary material for: A comparison between bacterial cultivation and 16S rRNA next generation sequencing approaches for analysis of bacteria in urine and cerebrospinal fluid samples
Source: PLoS One. 2026 Jun 25;21(6):e0350939. doi: 10.1371/journal.pone.0350939 (PMC13298949; doi:10.1371/journal.pone.0350939)
Supplement: S9 Table — (DOCX) [file pone.0350939.s009.docx]

**S9 Table:** The most common microorganisms obtained by NGS DNA sequence analysis from CSF samples that showed negative bacterial growth, classified based on genus.

| **Bacterial genus** | **Total reads** | **Frequency (Sample Number)** |
| --- | --- | --- |
| *Variovorax* | 1074 | 8 |
| *Pseudomonas* | 805 | 9 |
| *Sphingomonas* | 655 | 9 |
| *Methylobacterium* | 392 | 7 |
| *Enterobacter* | 340 | 9 |
| *Escherichia* | 282 | 9 |
| *Staphylococcus* | 255 | 9 |
| *Lactobacillus* | 255 | 6 |
| *Polaromonas* | 235 | 7 |
| *Delftia* | 214 | 8 |
| *Paucibacter* | 199 | 5 |
| *Rickettsia* | 187 | 8 |
| *Prevotella* | 139 | 5 |
| *Chryseobacterium* | 126 | 3 |
| *Gardnerella* | 116 | 3 |
| *Serratia* | 114 | 9 |
| *Acinetobacter* | 94 | 9 |
| *Rathayibacter* | 90 | 4 |
| *Providencia* | 86 | 8 |
| *Corynebacterium* | 81 | 8 |
| *Ureaplasma* | 77 | 1 |
| *Klebsiella* | 76 | 5 |
| *Stenotrophomonas* | 72 | 6 |
| *Faecalibacterium* | 65 | 6 |
| *Bacillus* | 64 | 8 |
| *Limnobacter* | 60 | 5 |
| *Streptococcus* | 59 | 6 |
| *Nevskia* | 50 | 7 |
| *Pediococcus* | 48 | 4 |
| *Erwinia* | 42 | 3 |
| *Ruminococcus* | 40 | 5 |
| *Blautia* | 39 | 5 |
| *Azohydromonas* | 39 | 4 |
| *Peptoniphilus* | 38 | 5 |
| *Veillonella* | 38 | 3 |
| *Enterococcus* | 36 | 5 |
| *Bacteroides* | 35 | 5 |
| *Streptomyces* | 34 | 8 |
| *Tolumonas* | 33 | 5 |
| *Bifidobacterium* | 33 | 3 |
| *Micrococcus* | 32 | 3 |
| *Clostridium* | 29 | 6 |
