# Supplementary material for: A comparison between bacterial cultivation and 16S rRNA next generation sequencing approaches for analysis of bacteria in urine and cerebrospinal fluid samples
Source: PLoS One. 2026 Jun 25;21(6):e0350939. doi: 10.1371/journal.pone.0350939 (PMC13298949; doi:10.1371/journal.pone.0350939)
Supplement: S10 Table — (DOCX) [file pone.0350939.s010.docx]

**S10 Table:** The most common microorganisms obtained by NGS DNA sequence analysis from CSF samples that showed negative bacterial growth, classified based on species

| **Bacterial species** | **Total reads** | **Frequency (Sample Number)** |
| --- | --- | --- |
| *Variovorax paradoxus* | 967 | 8 |
| *Pseudomonas xanthomarina* | 463 | 2 |
| *Sphingomonas oligophenolica* | 288 | 8 |
| *Sphingomonas echinoides* | 241 | 6 |
| *Escherichia coli* | 234 | 9 |
| *Polaromonas jejuensis* | 218 | 7 |
| *Delftia lacustris* | 214 | 8 |
| *Methylobacterium marchantiae* | 195 | 5 |
| *Lactobacillus iners* | 151 | 5 |
| *Pseudomonas stutzeri* | 136 | 1 |
| *Gardnerella vaginalis* | 116 | 3 |
| *Methylobacterium adhaesivum* | 106 | 6 |
| *Variovorax boronicumulans* | 97 | 5 |
| *Enterobacter hormaechei* | 77 | 6 |
| *Prevotella copri* | 75 | 3 |
| *Enterobacter amnigenus* | 67 | 6 |
| *Enterobacter nickellidurans* | 64 | 4 |
| *Providencia rettgeri* | 59 | 8 |
| *Ureaplasma parvum* | 59 | 1 |
| *Enterobacter ludwigii* | 51 | 6 |
| *Ralstonia pickettii* | 47 | 7 |
| *Chryseobacterium bovis* | 47 | 1 |
| *Rathayibacter caricis* | 45 | 4 |
| *Klebsiella oxytoca* | 44 | 2 |
| *Limnobacter thiooxidans* | 38 | 4 |
| *Erwinia billingiae* | 37 | 2 |
| *Pediococcus stilesii* | 35 | 4 |
| *Tolumonas auensis* | 33 | 5 |
| *Nevskia ramosa* | 30 | 6 |
| *Ralstonia detusculanense* | 30 | 4 |
| *Stenotrophomonas pavanii* | 29 | 4 |
| *Stenotrophomonas geniculata* | 29 | 3 |
| *Veillonella montpellierensis* | 28 | 3 |
| *Enterobacter asburiae* | 27 | 1 |
| *Acinetobacter johnsonii* | 26 | 5 |
| *Micrococcus yunnanensis* | 25 | 3 |
| *Rathayibacter tritici* | 24 | 4 |
| *Lactobacillus ultunensis* | 23 | 4 |
| *Pseudomonas chloritidismutans* | 23 | 3 |
| *Bacillus litoralis* | 22 | 6 |
| *Acinetobacter tjernbergiae* | 22 | 5 |
| *Lactobacillus taiwanensis* | 21 | 4 |
